# Getting the timing right. Autistic adolescents reflect on the value of an early diagnosis

**DOI:** 10.3389/fpsyt.2026.1735842

**Published:** 2026-05-14

**Authors:** Gert-Jan Vanaken, Ilse Noens, Jean Steyaert, Kristien Hens

**Affiliations:** 1 Centre for Ethics, Department of Philosophy, University of Antwerp, Antwerp, Belgium; 2 Parenting and Special Education Research Unit, Faculty of Psychology and Educational Sciences, KU Leuven, Leuven, Belgium; 3 Leuven Autism Research (LAuRes), KU Leuven, Leuven, Belgium; 4 Centre for Developmental Psychiatry, Department of Neurosciences, KU Leuven, Leuven, Belgium

**Keywords:** adolescents, autism, ethics, interviews, neurodiversity, timing of diagnosis

## Abstract

**Introduction:**

In Western countries, autism diagnoses are increasingly assigned in the first years of life. But is earlier necessarily better? Despite potential benefits, autistic infants and toddlers cannot participate in these discussions. In the ethical debate on early autism diagnosis, this raises tensions between parental duties and rights, and the child’s developing autonomy.

**Methods:**

To mend the lack of autistic voices in this debate, we queried a diverse group of 18 autistic adolescents (aged 16–18). In a set of indepth interviews, we explored their experiences of their autism diagnosis, and their views on the ideal timing of such a diagnosis, if at all.

**Results:**

Using the QUAGOL data-analysis method, we developed three themes: (1) (Not) feeling different, (2) Drawing up the balance of the label’s value, and (3) Getting the timing right. Adolescents experiencing most difficulties in navigating the neurotypical world also seemed to value the diagnostic label most, and vice versa. Nevertheless, nearly all adolescents favored a relatively early diagnosis and early disclosure thereof—not necessarily in infancy, but early enough to enable timely support for both themselves and their parents. Crucially, adolescents emphasized that such early support should be personalized, readily available and neurodiversity-affirmative to make early diagnosis truly worthwhile.

**Discussion:**

Our data did not corroborate any presumed clash of interests between parents and autistic children. Consequently, we suggest moving this ethical debate away from a discourse based on individual rights or interests toward a relational, care ethics approach.

## Introduction

Being assigned an autism diagnosis can be a life-changing event. It may influence how autistic^1^ people view themselves, how relevant others perceive and interact with them, and how they navigate public life (1). Despite the label’s impact, an autism diagnosis is currently, in Western countries, most often assigned to toddlers and children; people who cannot (easily) voice their opinion on whether it is desirable to obtain such a lifelong diagnosis (2, 3). Of course, caregivers and diagnosticians can have valid reasons to seek and assign a diagnosis for a young child. For many caregivers, for example, an early^2^ diagnosis may help to improve their understanding of their child’s behavior, to parent in a more attuned way and to access autism-specific care (5, 6).

But despite such potential advantages, ethical tensions do remain concerning such early autism diagnoses. For example, the aim to do good for the child by assigning a timely diagnosis and giving access to appropriate care, can conflict with the aim to respect the child’s developing autonomy abilities, i.e. to have a say in receiving a lifelong diagnostic label (7, 8). Additionally, since the upsurge of the neurodiversity and autistic self-advocacy movement, newly discussed advantages of a diagnostic label include affirmative identity work, i.e. embracing and accommodating one’s autistic characteristics, and finding peer support in the autistic community (9). Yet, the extent to which these identity and community issues play out in the context of early diagnostics is not well studied, making it difficult to add them to the ethical equation.

Also, even when there are good intentions to seek beneficial outcomes for the child in case, assigning an autism diagnosis can also lead to disadvantageous labelling effects. Expectations for the child might be unduly lowered, stereotypical views of autism might guide the child’s upbringing and ableist early interventions aimed at normalizing the child’s behavior might be pursued (10).

These tensions are central to ongoing debates about the ethics of early autism detection and diagnosis (8, 11). Such debates are likely to gain further prominence as efforts continue to identify and implement reliable screening parameters for autism in infants and toddlers (2, 12). Until recently, empirical work on the ethics of early autism care mostly engaged with academic, clinical and parental perspectives, much less with autistic people’s actual experiences receiving an early diagnosis (8, 11). With the current in-depth interview study, we aim to address this gap by gaining a deeper understanding of how children experience being assigned an autism diagnostic label.

Two reasons drove our choice to query adolescents between the ages of 16 and 18, instead of younger children. First, to allow for a diversity of experiences, we aimed for a sample including both relatively early diagnosed (i.e. in childhood, cf. infra) and later diagnosed (i.e. in adolescence) interviewees. Second, inspired by previous work in this field (e.g. 13), we anticipated that (autistic) identity formation might play a role in autistic youth’s evaluation of the appropriate timing of a diagnosis. Compared to younger children, we anticipated that adolescents were better positioned to reflect on this matter.

Our research questions were as follows.

How do autistic adolescents experience autism and their autism diagnosis? What do they consider a good timing for diagnosis, if at all, and why?

### Literature overview

Previous in-depth interview studies with autistic adolescents have tapped into questions related to ours although these studies have generally not been explicitly situated in the debate on the ethics of early autism diagnosis.

In one of the first publications of its kind, Huws and Jones (14) described how the diagnostic label enabled autistic adolescents to review and understand past life experiences. The authors called this ‘biographical disruption’: receiving and knowing about their diagnosis was a turning point in these adolescents’ life as it changed how they viewed themselves, how others perceived them and how their educational and professional opportunities developed.

Mogensen and Mason (15) reported on the effects of an autism diagnostic label on adolescents’ sense of self-identity, which ranged from feeling oppressed to feeling proud about being autistic. Based on their interviews, the authors suggested that these varying effects of diagnostic labelling are not random. Adolescents tended to experience their diagnosis as advantageous when it helped them understand themselves and gain control over their lives. For example, after disclosure of the diagnosis, adolescents reported that learning and self-managing to structure their days made them fare better. The opposite tended to be true when the autism diagnosis led to reduced control over their lives, for example when their diagnosis was used as an argument to change schools against their will.

The existing literature also touches upon autism-related identity formation in adolescents. In a systematic review and meta-synthesis of qualitative research with youth with a psychiatric or developmental diagnosis, O’Connor and colleagues (13) found that autistic adolescents differ in such identity-related aspects from adolescents with chronic mental health conditions as a primary diagnosis. According to this analysis, autistic adolescents, compared to other clinical groups, appreciated their diagnostic label more positively, and expressed a preference for obtaining their diagnosis earlier in life.

Yet, being autistic is not unequivocally understood in positive terms. Jones et al. (16) described a ‘state of incongruence’ among their interviewees ‘wanting to reject the part of their diagnosis that they disliked or perceived as socially unacceptable while simultaneously maintaining the parts of their diagnosis that made them unique or talented’ (16, p. 1493).

Reviewers of qualitative literature with autistic youth have identified several methodological challenges and shortcomings. Previously, intellectually disabled -and to a lesser extent female- participants have been underrepresented (17, 18; for a notable exception, see 19).

Drawing on a meta-synthesis of interview studies with autistic minors, Williams et al. (18) put forward three additional points of critique. First, in multi-respondent studies, the voices of autistic minors tended to be overshadowed by those of caregivers and clinicians. Second, interviewees’ diverging experiences and opinions have been underreported in favor of a single, coherent storyline. And third, autistic youth’s contextual variables, such as educational settings, have often been insufficiently described, limiting the interpretability of findings.

Additionally, McLaughlin and Rafferty (20) critiqued the tendency of researchers to interpret autistic youth’s stories primarily through a clinical lens that reaffirmed their autism diagnosis, rather than interpreting these stories as proper experiences with intrinsic, non-clinical value.

The present study presented is vulnerable to such critiques too, yet we have taken these issues to heart when designing and conducting this study. The write-up is based on COREQ-32 criteria (21) on reporting qualitative research and Van Schalkwyk and Dewinter’s (22) guidelines for the autism field in particular.

## Methods

### Recruitment and participants

We opted for semi-structured in-depth interviews with autistic adolescents between the ages of 16 and 18. We hypothesized that this age range would allow us to reflect on the value and timing of an autism diagnosis, including related identity formation issues.

Participants were recruited via the diagnostic database of the Expertise Centre for Autism embedded within the University Hospitals of Leuven, Belgium. We extracted a list of all people in the database between the ages of 16 and 18, excluding those with a total intelligence quotient (TIQ) below 60 and/or with a formally diagnosed language disorder. We considered these exclusion criteria relevant for participation in a written or spoken in-depth interview on this topic.

We selected potential participants from the database list -taking into account the rules mentioned below- and invited them to participate by calling their parents. Upon invitation, three potential participants declined to participate. **Table 1** provides an overview of the characteristics of the 18 included interviewees.

**Table 1:** Participant characteristics; F2F, face-to-face.

Inter-viewee n°	Age	Age at diagnosis	Age at disclosure	Gender	Total IQ	Educational setting	Autistic family members	Interview duration	Interview modality
1	16	9	9	female	80-120	regular	none reported	34	F2F
2	16	8	8	female	80-120	regular	3 siblings	39	F2F
3	17	4	6 (estimation)	male	80-120	regular (currently)	2 siblings	36	F2F
4	17	3	5 (estimation)	male	80-120	special	none reported	50	F2F
5	18	7	7	male	80-120	regular	none reported	68	F2F
6	17	9	12	male	80-120	special	none reported	52	F2F
7	17	14	14	male	>120	regular	none reported	43	videocall
8	17	14	14	male	80-120	regular	none reported	45	videocall
9	18	15	15	female	>120	regular higher education	suspects 3 siblings to be undiagnosed autistics	82	videocall
10	16	12	12	male	>120	special	none reported	67	videocall
11	16	14	14	male	>120	regular	none reported	68	videocall
12	17	15	15	male	80-120	regular	1 sibling	54	videocall
13	16	4	no response	female	<80	special	none reported	63	F2F
14	16	3	no response	male	<80	special	none reported	39	F2F
15	16	15	15	female	<80	special	none reported	66	videocall
16	16	5	15	male	<80	regular (currently)	none reported	58	F2F
17	17	13	13	female	<80	regular	1 sibling	57	F2F
18	17	6	6	male	<80	special	none reported	39	F2F

To achieve a balanced interview sample, we set out to include an *a priori* number of 18 interviewees in total, of which at least six with a borderline TIQ (60-80). We predefined a 3:1 male to female ratio and aimed for a 1:1 ratio in terms of timing of the autism diagnosis under the age of 10, or above the age of 12. We applied this age-based distinction in line with our research questions: individuals diagnosed after the age of 12 were likely to have been aware of -and possibly involved in- the decision to initiate a diagnostic assessment, whereas this was less likely for those diagnosed before age 10. This was an *a priori* assumption and we did not include a specific interview question to query about this in more detail because we were more interested in post-diagnostic experiences, rather than in discussion leading up to a diagnostic assessment.

Beyond age at diagnosis, also the age at disclosure is clearly important when documenting people’s experience of receiving the label. Before starting the interviews, we were unaware of interviewees’ age at disclosure, i.e. the age at which they were first told they were diagnosed autistic. Upon asking during the interview, we learned that all eight adolescents diagnosed after age 12 learned about their diagnosis right after the assessment process. For those diagnosed under age 10, we noted more variation. For four of them, disclosure followed assessment straightaway. Two only learned about it at age 12 and 15 respectively. For those diagnosed earliest, i.e. at age 3 or 4, data were less reliable: two of them recalled being told about their diagnosis at age 5 and 6 respectively, but weren’t entirely sure; two did not respond to this question.

The Ethics Committee of the University Hospitals Leuven provided ethical clearance (study number S62947). We collected informed assent from the participating adolescents and informed consent from their parents before the interview. Additional information regarding the patient database, the consent procedures, reporting of IQ scores, and the regional educational context can be found in the **Supplementary Material**.

### Data collection

For reasons of accessibility, participants could choose between three interview modalities: (1) a face-to-face conversation at a place of their preference, (2) an online video call, and (3) an online, written chat via a secured messaging platform. Eleven adolescents chose face-to-face conversation; all chose their home as their preferred place. Seven opted for a videocall; no one opted for written communication. All interviews were conducted in Dutch, except for interview 13 taking place in English. Data collection took place between August 2019 and September 2020.

This topic list was developed by an interdisciplinary team (GJV, JS, KH) with complementary experience in qualitative research, (bio)ethics and clinical practice with autistic people. We did not consult autistic community members explicitly on this point. The topic list was piloted and approved after two mock interviews with autistic youth matching our inclusion criteria; this data was not used for analysis. The final topic list can be consulted in the **Supplementary Material**.

Interviewees received the topic list a couple of days beforehand to increase predictability and provide preparatory and processing time. During the face-to-face interviews, this topic list was visible on the table; during video calls, we asked participants to have the printed list nearby or at hand in another window on their computer. This way, the topic list functioned as a visual support providing structure to the conversation.

All interviews were conducted by the first author. At the time of data collection, he was a male 27-year-old PhD candidate with a medical degree and experience in both clinical and academic interviewing. Master students accompanied the main interviewer on some occasions. None of the interviewees knew the interviewers or vice versa. In one interview, the participants’ mother remained present and assisted the interviewee with her consent (Interview 15).

The interviews lasted between 34 and 82 minutes, averaging 55 minutes. Live interviews were audio recorded; online interviews were both audio and video recorded. All interviews were transcribed literally in full, assisted by *f4transkript* software (23). Names and other identifiers were pseudonymized during the transcription process. We made field notes, but only to keep track of the conversation during the interview. These notes were not included in the analysis. Transcripts were not returned to participants for comment or correction, nor did we do so with our findings.

### Data analysis and positionality

We employed the Qualitative Analysis Guide of Leuven (QUAGOL) to analyze our data (24). The QUAGOL guide is a comprehensive and systematic approach to qualitative data analysis mainly embedded within Grounded Theory. It consists of two parts. First, there is a preparatory, inductive phase leading up to a list of codes. Second, there is a deductive phase of the actual coding of the transcripts based on the list of data-driven codes developed before. Based on the coded fragments, codes are developed in well-described concepts which are then structured in themes and subthemes. For this current study, we kept McLaughlin and Rafferty’s call in mind (20) not to view the interview data merely through the medical lens of autism’s diagnostic criteria. We avoided interpreting the adolescents’ experiences merely as symptoms confirming the diagnosis. Rather, we intended to start with a blank sheet, to accept the views of the participants as those of experts of their own lives, and then to reflect critically on those views. The quotes cited below are direct translations from the original transcripts in Dutch.

Theoretically, we commit to a neurodiversity perspective on autism research (25), embedded within a critical realist philosophy of science (26, 27). A more detailed description of our application of the QUAGOL guide, our positionality as researchers and our theoretical stances have been reported elsewhere (6, pp. 4134–4135).

## Results

As described, our aim was to investigate how autistic adolescents experience autism and their autism diagnosis, and how they think about the appropriate timing of such a diagnosis, if such an ideal timing exists at all. Below, we describe three themes we constructed throughout the analysis. [1] *(Not) feeling different*, [2] *Drawing up the balance of the label’s value*, [3] *Getting the timing right.* Admittedly, at first sight, only the third theme deals directly with adolescents’ views on diagnostic timing. Yet, as will become clear, the first two themes are important to fully understanding and appreciating adolescents’ opinions on the value of a timely autism diagnosis.

### Theme 1: (not) feeling different

This theme zooms in on how participating adolescents experienced autism and (potential) autism-related differences from the norm. A first group of adolescents (Interviews 1, 2, 8, 12, 13, 15, 16, 18) shared experiences about not feeling particularly ‘*different’* compared to others or even feeling plainly ‘*similar to other people*’ (Interview 18). Whether someone feels different or not, obviously depends on who they are comparing themselves with. Generally, adolescents made these comparisons with people they engage with in their daily lives and not so much with an abstract idea of the typical 16- to 18-year-old. For example, when we asked a girl who attends special education whether using the quiet playground at school made her stand out compared to her classmates, she responded:

‘Me and my friends, we all go there’ (Interview 15).

In other words, certain autism-related behaviors and choices do not manifest as out-of-the-ordinary in this girl’s school setting. Rather, these features are shared with peers and relatively well accommodated in this case. As a consequence, for this first group of adolescents, the experience of being autistic largely operates in the background of their lives. In this respect, one boy said,

‘I don’t think about it all the time, in fact, I hardly ever think about autism’ (Interview 8).

Despite such expressions of relative indifference toward autism, these adolescents do name certain experiences as autistic experiences.

Autism is part of who I am, but in daily life, it does not occupy me, because I simply don’t feel bothered. Except for one thing: my planning. I really cannot stand it when things I like to know beforehand what is going to happen, and I find it difficult when things change last minute (Interview 2).

Another girl said:

Sometimes I forget I have autism, but when I am in a situation where I get really angry, then I turn into another person. Then, sometimes, I think, oh this might be my autism coming to the surface (Interview 1).

Responding to the question of whether she experiences autism more explicitly in certain situations compared to others, a third girl responded as follows:

Yes, when there are appointments I am not prepared for. For example, I had to go to an interview that I forgot about. And suddenly mom entered the classroom, and we had to leave. I didn’t know where we were going. (…) that felt overwhelming (Interview 15).

This way, these adolescents seemingly understand autism as (negatively valued) manifestations of person-environmental mismatch or emotional loss-of-control, and not so much as a given that also shapes neutral or more positively valued experiences. For example, this last girl also talked about how she could spend many hours at her drawing desk enjoying being absorbed by the activity -described favorably by autistic scholars as ‘flow states’ (28). Yet, the interviewed girl explicitly stated this state of flow had nothing to do with autism.

#### Difference

For other adolescents, autism is more woven into their daily lives and appears more systematically at the forefront of their thinking (Interviews 3, 4, 7, 9, 10, 11, 17).

‘Psychologically, it is a big part of who you are, because every day and every week, you have to deal with that and with how you behave’ (Interview 4).

These adolescents reported feelings of autism-related differences more explicitly compared to peers. One boy said,

‘I’ve been having this feeling all my life. (…) I have always known I was different; the label just confirms that I am different, it confirms something I already knew’ (Interview 7).

For most interviewees here, this experience of feeling different is a longstanding one that, indeed, predates their awareness of their autism diagnosis and that was expected to continue over time. Consequently, receiving their autism diagnosis, or being told about it, was something relatively unsurprising for this subgroup of adolescents.

Autism will always be part of who I am, and that influences all my choices. Not necessarily in a negative way. But it will always influence me, because my way of reasoning is just like that, because I have ASD [sic]. And I don’t think that will change anytime soon (Interview 9).

### Theme 2: drawing up the balance on the label’s value

In addition to describing autism and sharing experiences of autism-related differences, adolescents talked about how they perceived the value, if any, of their autism diagnostic label. Two categories of responses emerged from our analysis: (1) the direct value of the label to the youth themselves, (2) the indirect value of the label mediated through other people. The former is of course dependent on *knowing* one is autistic and thus only follows after their diagnosis was disclosed to them.

#### The label’s direct value for oneself

Relating to the direct value of the diagnostic label, two positions stood out: some adolescents experienced little additional value from the diagnosis for themselves, while others did. In terms of group composition, there is overlap between those experiencing little additional value (Interviews 1, 2, 8, 13, 14, 15, 18), and those feeling not very different compared to peers as discussed in the second theme.

One girl, growing up in a family with two autistic brothers and two autistic parents, phrased this position of little additional value as follows:

‘I don’t experience much difference as it [i.e. autism] does not bother me. Except for missing out on the extra support in elementary school, it would not have bothered me if I never got the diagnosis’ (Interview 2).

This echoes the finding from Interview 12, where the girl in case said that she was fine with receiving her diagnosis at age 14 since accommodations were already in place at home for her autistic brother.

Two boys clearly stated that they did not care at all about having received the diagnosis (Interviews 14, 18).

Three girls (Interviews 12, 13, 15) indicated here that for them it is ‘*good to know*’ about their autism diagnosis, although explaining why that was the case seemed difficult. In one of those interviews, the mother of this girl, who was present in the room, made a clarifying addition:

‘In fact, that [autism diagnosis] was not that important for us. But for school it was’. The adolescent girl added: ‘Oh yes, now I remember, it was for that internship,’ (Interview 15)

referring to the possibility of engaging an additional mentor reserved for those interns with a formal autism diagnosis. For context, this girl received a diagnosis of intellectual disability earlier in life. This first diagnosis already guided her parents, other family members, teachers, and the girl herself to a large extent in organizing her life in an accommodating way. At the point of interviewing, the additional and more recent autism diagnosis only had little direct added value.

A second group of adolescents talked about how they had experienced direct value in having received and knowing about their autism diagnosis (Interviews 3, 7, 9, 10, 17). Here, they mentioned how the label provided recognition for experienced differences, exculpated undesired behaviors, explained atypicalities, and helped to position themselves toward these differences, behaviors, and atypicalities. Below, we elaborate on the latter two functions of the label: explaining atypicalities and positioning oneself.

According to several adolescents, knowing they are autistic helped them to ‘explain’, for example, why they think differently, find joy in things others might not, and get upset by situations deemed unremarkable by others. Here, adolescents understood ‘explanation’ not so much as in ‘explaining something to others’^3^, but more as an individual process. An 18-year-old girl phrased it as follows:

I wasn’t surprised to hear I had autism; it rather explained things to me, my behavior and my thoughts. It explained why I react in certain ways in some situations. And why some people can handle these situations better than me (Interview 9).

The diagnostic label gives these adolescents a frame of reference to think about their current and past selves. One girl said for example:

‘I used to be very fixated on wearing the right gloves, and now I realize in such situations: alright that is because of autism’ (Interview 17).

She explained afterward that this insight did not necessarily make her change those behaviors, but it helped her to experience behaviors in a more accepting, welcoming way.

In addition to explaining difference, knowing about their diagnostic label also helped some youth to position themselves in relation to their experiences of difference from the norm. This positioning can mean being more accepting of oneself, but also deliberately choosing moments to blend in with a (neurotypical-dominated) group, or hiding autistic features more systematically. One boy who was quite at peace with his autism diagnosis put it this way:

If I would have known earlier which things are different for me, then I could have paid more attention maybe to those aspects, without changing myself into someone else entirely different of course, which is not possible. In that sense, the label could help, because you know better in what respect you are different (…) But you cannot focus on being different too much, because if you think that something is wrong with you and you don’t accept yourself, friends won’t do so either. That’s a big step, realizing there isn’t something wrong with you, but that you are just different. And that there are small things you can do about that, but that you should not try to change yourself fundamentally, because that doesn’t work at all (Interview 7).

For another adolescent, this ‘positioning oneself’ translated into attempts to blend in with non-autistic peers and to hide autistic features actively (Interview 16).

On the one hand, I would rather just not have autism. But on the other hand, I am happy to know I have it, so I can be aware of how I behave and how I talk. I try to laugh less exaggeratedly, just trying to laugh normally. Making sure I don’t say any stupid stuff.

For one 17-year-old who only received his diagnosis three years before, the diagnostic label made him position himself much more reluctantly in social contexts, saying he feels

‘frightened that others consider him a weirdo [ … ], while four, five years ago I used to approach anyone without thinking things over’ (Interview 11).

#### The label’s indirect value mediated via others

Beyond direct, valuable consequences of receiving the diagnostic label, interviewees also brought up a range of effects mediated by others. For example, an autism diagnosis provided them with an official justification to access specific care services and accommodations. Also, when parents, teachers, and friends are aware a child is autistic, our interviewees thought it would be easier for these people to take into account particular autistic features and needs during interactions. It was hypothesized that such awareness would reduce misunderstandings, frustrations, and conflicts.

Yet, making others interact in such more autism-accepting ways generally requires adolescents to disclose their diagnosis explicitly to their interlocutors. In the case of our interviewees, parents were always aware of the diagnosis, as all participants received their autism diagnosis as minors with their parents taking a leading role in initiating the assessment. Almost all adolescents indicated they found it understandable that their parents initiated a diagnostic assessment at a certain point in time, even though obtaining such a diagnosis potentially has long-lasting impacts on their life. The following quotes illustrate this position.

‘I can imagine that as a parent, you want to know what your child has. That’s normal’ (Interview 18).

‘If parents think that something is wrong, then they have to know what is going on, so they don’t always worry’ (Interview 8).

‘This way they can change how they behave and raise their child and maybe look out for the right school’ (Interview 5).

When it comes to people other than parents, adolescents talked about the balancing act of disclosing their diagnosis. According to the respondents, the main aim of disclosing one’s diagnosis is for the other person to take into account autistic characteristics and needs, although this does not always happen.

One boy said:

Okay, you can say this to people and hope they take it into account, but at the same time you don’t always want to send this message to the world, as in reality, people don’t always do take it into account (Interview 7).

Without prompts in this direction, many adolescents relied here on a distinction between ‘taking into account’ autistic features and needs, and ‘being treated differently’ because of them. The latter option clearly had a more negative connotation. One girl shared a telling anecdote which took place during her internship as a caregiver in a hospital (Interview 17).

The people really saw me as “the intern with autism”, not just an intern. (…) The head nurse told me, “I come specifically for you to the ward to help you”. But, for me, a regular nurse would have been just fine. (…) I don’t need a head nurse to teach me just because I have autism. I just wanted to be treated as an equal. Sometimes I will face some difficulties, but then I will just ask for help, head nurse or not.

This episode illustrates how ‘being treated differently’ connects to paternalistic interaction patterns and stereotypical, negatively charged or simply incorrect views of autism, on behalf of the other party. Moreover, as this girl indicated, diagnostic disclosure to third parties can lead to autism becoming one’s ‘primary identity’ (‘the internee with autism’) in the eyes of the other, even when this does not match with the adolescent’s perception.

Faced with such potential pitfalls, most teens share their diagnosis only when they feel it is necessary to resolve a particular tension, such as when they feel they will be misunderstood or considered rude if they share their thoughts in a straightforward way (Interview 3, 9, 17). In situations and contexts where they can blend in or pass as non-autistic (Interviews 1, 2, 9, 16, 17), or when their environment is already well aware of their autism diagnosis (Interviews 4, 6, 13, 14, 15, 18), disclosure is considered less urgent. Some adolescents shared experiences of unwanted disclosure of their diagnosis, for example when having to show a justificatory document in the classroom to obtain accommodations, all while classmates were observing.

Finally, and this was rather an exception, one girl shared a story of sharing her diagnosis with her boyfriend, not so much out of an urgent need to resolve tension, but because she trusted this information would be in good hands (Interview 9).

### Theme 3: getting the timing right

When asked about an adequate timing of an autism diagnosis, overall, the interviewees expressed themselves in favor of a relatively early autism diagnosis. For the interviewees, a ‘relatively early’ diagnosis meant that the diagnosis would be assigned at a moment when they themselves cannot fully understand the diagnosis and its implications. This boiled down to a moment under the age of six to eight years, depending on the child. One girl diagnosed aged 14 said, exceptionally, that the timing was just right for her. In the subsections below, we first discuss findings related to receiving an early diagnosis irrespective of whether the child has been told about it. This subsection mainly discusses the role of parents and other caregivers. The second subsection deals with issues related to the child being actively aware of their autism diagnosis at a young age. This latter issue is thus reliant on diagnostic disclosure toward the child in case.

#### Receiving an early diagnosis

According to many interviewees, a first potential advantage of a relatively early diagnosis is to help create more fitting and supportive environments in early life to grow up in, both at school and at home, even if they are not aware of the diagnosis themselves at that point. Several interviewees explained this as follows. One adolescent diagnosed at age 12 argued this was

‘way too late because it can be found much earlier’. (…) And if they would have known it in elementary school, they maybe could have taken this a bit into account and things would have been a whole lot easier’ (Interview 10).

Another boy, diagnosed at age 14, said:

‘the earlier you get your diagnosis, the bigger part of your life people can take this into account’ (Interview 7).

A girl, diagnosed at age 8, hypothesized that not having a diagnosis in elementary school would lead to ‘*missing out on opportunities to get additional support’*, which was something she appreciated when in elementary school (Interview 2). A girl, diagnosed at age 15, explained that

‘certain things have gone particularly wrong, at school and at home, because I have autism. But if I had reacted differently at that time because I knew I had autism, we could have avoided some trouble’ (Interview 9).

Most adolescents were also convinced that a relatively early diagnosis could be helpful or even necessary for their family members, especially their parents. Here, arguments like those in the previous sub-theme on the “indirect value of the autism label” were echoed. One participant said that:

If autism is diagnosed early, the advantage for parents is that they know better what to do. If they wait until adolescence for example, then things will get complicated. So, I am not sure if there is an ideal age for diagnosis, but at least before adolescence (Interview 6).

Next to acknowledging their parents’ need to ‘know what is going on with their child’ and ‘how to deal with that’, some adolescents also recognize the potential difficulties for parents in such situations.

‘I think for every parent, it is useful to know whether your child has autism, but this is also just difficult because later on, you will have to tell your son or daughter’ (Interview 16).

Another boy considered that it could be:

Emotionally rough for parents [when they hear about the diagnosis] because they realize they will need to care more for their child than expected. So that is maybe a disadvantage of an early diagnosis, but even though it can be rough, earlier is better, at least you know what to do with your child then (Interview 6).

One girl also pointed to parents’ balancing act of considering an autism diagnostic assessment, knowing that mainstream views of autism are fairly negative.

I get that parents do not want their child to have an autism diagnosis, because people sometimes have a wrong image of what it is, while for some who have autism, it does not stand out at all, or they only have some small things that make you notice. But people sometimes think it is something severe and I think that scares parents. They do not want their child to have it because other people think badly about it (Interview 2).

One girl held a minority opinion saying that for her personally, she couldn’t think of advantages to receiving her diagnosis before she did at age 14. Notably, her brother was autistic too.

‘I don’t know [whether it would be advantageous to have an earlier diagnosis]. We always had sufficient structure at home’ (Interview 12).

In the same vein, she commented that she could see why a diagnosis would be more helpful for other people earlier in their lives. But for her, the diagnosis at age 14 was just fine.

#### Knowing one is autistic early on

Next to indirect advantages mediated via external support or their parents, several adolescents also point to more direct advantages of a relatively early diagnosis for themselves. Knowing they are autistic themselves as soon as reasonably possible could help self-exculpating and explain atypical characteristics and help position themselves toward these characteristics (Interviews 3, 4, 7, 9, 10, 16).

I think it is best to know as early as possible, because, yeah, you have to grow up with it, you have to learn to live with autism. And growing up, that is what you do when you are young, not when you are fifty. [ … ] Earlier is better because otherwise, you might suffer for years not knowing what is going on, making bad choices in your life. With an early diagnosis, you can grow up with that, learn what it is, and deal with it (Interview 3).

However, for a child to consciously engage with their autistic characteristics, the child must of course be informed of the diagnosis. It is important to distinguish between the timing of diagnosis in clinical practice and the timing of disclosing that information to the child in case.

Once the diagnosis can be established properly when the characteristics can be noticed, then I would just go for it. The child doesn’t need to know straight away, but at least the school and parents can know it this way (Interview 10).

Another boy added here,

‘as soon as the child can understand it, then they should be told (…), I really believe that is the best thing to do’ (Interview 4).

Adolescents disagreed about the right age for explicit communication of the diagnosis. For some, it is around seven or eight years, for others around the start of secondary school, i.e. around twelve years of age, depending on the child’s abilities and needs to appreciate this knowledge. More explicitly, however, several adolescents pointed out that waiting too long or keeping the diagnosis a secret is undesirable and can lead to potentially distressing experiences at the time of delayed disclosure. Two adolescents experienced such a delayed disclosure (Interviews 6 and 16, see **Table 1**). One of them, a 16-year-old who was diagnosed at age 5, said the following:

‘I only learned about it last year. (…) Then they said it, and of course, it throws you off completely. (…) I was appalled; I did not expect this at all’ (Interview 16).

Apart from these positions that stressed the advantages of a relatively early diagnosis, we also documented some opposite views. Two participants raised doubts about receiving their diagnosis earlier than they did right now, as they

‘did not feel different from others’ (Interview 14), or feared to be *‘treated differently in elementary school’* (Interview 8).

Also, two adolescents took a position of indifference (Interviews 13, 15) stating they did not really care about the timing of their diagnosis, as they did not care much about their diagnosis at all.

For the interviewees, a ‘relatively early’ diagnosis thus meant the diagnosis would be assigned at a moment at which they are not yet fully aware of it. Yet, in the research literature and clinical practice, the term ‘early’ detection and diagnosis of autism tends to be reserved for practices within the first three years of life (4). When we prompted our interviewees to reflect on assigning an autism diagnosis to children under the age of 3 in particular, they voiced some advantages, a few doubts and a range of conditions to take into account. The advantages mentioned were mainly iterations of arguments mentioned before on generating a more accommodating environment to grow up in, and on helping parents to find their way in raising an atypically developing child. In terms of doubts, one adolescent questioned whether such a very early diagnosis could be technically possible and reliable, and another one wondered what the label’s impact on a child would be in early life (Interviews 4 and 7).

#### Conditionality

The conditions that were discussed for valuable, early diagnostics brought some new elements to the fore, though. The first condition was that adolescents indicated that a diagnostic assessment should only be considered when the child displays some (potentially) autistic features. A second condition to favor such very early autism diagnoses is that post-diagnostic support should be possible and relevant. Assigning an autism diagnosis at a point in time when it is still unclear for parents and other caregivers how to engage with this knowledge adequately, was not supported by the adolescents who talked about this. Several aspects emerged when thinking about relevant post-diagnostic support following an early diagnosis. Support should be concrete and tailored to the specific needs and circumstances of each individual case.

They need to be able to say what is going on exactly, what autism does for this child in particular. Because if they say he has autism and just leave it at that, parents might start thinking: my child will act weirdly later. And parents might start spoiling their child and so (Interview 8).

Also, according to another adolescent, information about autism should be nuanced, not only focusing on deficits. He said that

‘an important aspect when assigning a diagnosis is to show there are not only downsides [to autism], but that the child also has been lucky in other respects’ (Interview 7).

A third conditional remark made by a few adolescents concerned the role of parents in the post-diagnostic period. Having supportive parents was not taken for granted by adolescents.

I think the value of an early diagnosis depends a lot on the parents. Personally, I know some people who will surely end a pregnancy when they would know their child would be autistic because they want a normal child per se. (…) But if you have supportive parents, who are eager to learn about autism, then I think such an early diagnosis could definitely help (Interview 9).

Therefore, parents should also be well-supported themselves to engage appropriately with their autistic child.

‘I think it would be a good step when there is a lot of guidance (for parents). Because clinicians cannot just tell parents: your child has ASD [sic] and then move to the next case’ (Interview 9).

To wrap up this Results section, here is a brief summary of the three themes. Those adolescents experiencing most difficulties in navigating the neurotypical world, also seemed to value the diagnostic label most, and vice versa. Nevertheless, nearly all adolescents favored a relatively early diagnosis and early disclosure—not necessarily in infancy, but early enough to enable timely support for both them and their parents. Crucially, adolescents emphasized that such early support should be personalized, readily available and neurodiversity-affirmative to make an early diagnosis worthwhile. Below, we will discuss these findings in relation to the existing literature and explore their implications for the ethical debate on early autism diagnosis.

## Discussion of the findings

We undertook this interview study to improve our understanding of how autistic adolescents experience autism and their autism diagnosis, and more specifically how they reflect on the ‘right’ timing of such a diagnosis. Below, we will first situate our findings within the existing body of literature. We will discuss potential reasons for differences in how some interviewees valued their diagnosis more than others and how this influenced their opinion on diagnostic timing. We will also discuss the role of parents within these value judgements. Second, we will turn our gaze beyond the particularities of the 18 adolescents we interviewed and discuss the implications of this study for the ethical debate on early autism diagnosis.

According to our interviewees, an autism diagnosis comes with benefits and harms, and adolescents reach different points of equilibrium in their value judgement. None of the interviewees actively resisted or disputed their diagnosis, but for some the diagnosis operated rather in the background of their lives, while for others it functioned clearly at the forefront. We cannot make causal statements here, but based on our analysis, it seemed that the age at which they received their diagnosis (before 10 or after 12 years of age) did not drive this distinction. However, the subgroup of interviewees for whom their diagnosis functioned more in the background (cf. Theme 1) attributed less value to receiving the diagnosis (cf. Theme 2) and receiving it early on (cf. Theme 3). Vice versa, the subgroup dealing more actively with their diagnosis at the point of interviewing, was more favorable to receiving a diagnosis and receiving it early in life.

Interviewees seemed to adhere more often to this first subgroup when they felt well-accommodated in their current life stage (e.g. Interviews 15 and 18), or when they could sufficiently ‘pass as normal’ in their social environments without too many negative implications (e.g. Interviews 1 and 2). Also, the presence of other autistic people such as family members and peers might help ‘normalizing’ autistic traits -in the sense of expanding what is viewed as normal- and offer pockets of connection and understanding, which made the relevance of receiving an (early) diagnosis less pressing. This latter interpretation hints at the distinction between vertical and horizontal identities, as described by Andrew Solomon (29). Vertical identities are those where parents and children share a similar identity characteristic (e.g. ethnicity), whereas horizontal identities are those where children differ from their parents in a significant respect and develop an identity based on a characteristic shared with chosen peers (e.g. queerness). Autistic identity can develop both vertically and horizontally. Yet, in our findings, having autistic family members did not feature as the main determinant for adolescents to put autism upfront in their identity descriptions. The extent to which adolescents experienced difficulties in navigating various neurotypical contexts seemed more relevant. For example, some adolescents had multiple autistic family members and this implied that family norms were already rather autism-inclusive. This reduced the need to have autistic identity functioning on the forefront of their lives. Whereas others who had autistic siblings too, did engage more actively with autism as identity characteristic, mainly as a tool for self-understanding and to advocate for accommodations that were not yet in place for them.

Our findings also relate to the conclusions of a recent systematic review looking at determinants of positive autistic identity development (9). Here, external autism acceptance and external support came out on top, while personal characteristics mattered less. Future work could look into the relation between the valence (positive, negative) of autistic identity and the extent to which this identity marker functions on the forefront or in the background of people’s lives.

### Timely, rather than early diagnostics

When asked about their views on diagnostic timing, none of the interviewees explicitly opposed a relatively early diagnosis, i.e. a diagnosis which is assigned before a child can be (fully) aware of its differences compared to peers. A few adolescents, though, added they were rather indifferent to their diagnosis, including its timing, while for others, the relevance of an early diagnosis stood out. The age at which they were diagnosed themselves (before 10 or after 12 years of age) did not seem to play a significant role in this respect. Originating in different lived experiences, opinions broadly converged. For example, participants diagnosed in childhood discussed how additional support at school was a helpful consequence of their diagnosis, whereas those diagnosed in adolescence reflected on the difficulties they experienced in primary school due to the lack of a formal diagnosis.

Adolescents’ arguments in favor of such a relatively early diagnosis echoed those mentioned when discussing the value of a diagnosis in general. But they expanded on two elements. First, a relatively early diagnosis might help generate better-accommodated environments to grow up in. Second, it might help them relate to and live with autism as soon as they are old enough to be informed about the diagnosis. These two elements corroborate some of the qualitative findings from a recent mixed-method study investigating the relationship between autistic college students’ age at which they learned about being autistic and their current levels of well-being (30). In this study, participants brought up ‘access to support’ and ‘increased self-awareness’ as key explanatory factors for the suggested association between learning they were autistic early on, and their current quality of life (ibid, p. 7). This is much in line with our findings. However, in a replication study with a larger and more diverse sample, the quantitative association between age of learning about diagnosis and adult quality of life did not hold (31). Instead, the authors suggested that it is less about ‘when’, but also about ‘how’ someone learns they are autistic, who tells them and which types of support are available afterward.

In our own study as well, adolescents phrased their support for a relatively early diagnosis in a conditional way. As presented in Theme 3, several interviewees underscored that the value of a timely diagnosis depends largely on what happens post-diagnostically. Establishing a diagnosis should first and foremost help address or prevent experienced problems of autistic children and their relatives. Assigning a diagnosis at a point where such concrete action cannot be implemented yet, has less value according to several adolescents. In this sense, speaking of a ‘timely’ rather than an early diagnosis might be more helpful.

### Supportive and well-supported parents

Next to the appropriate timing, the actions and attitudes of parents and clinicians are important to turn an early autism diagnosis into something valuable. Or, as we have put it before: supportive and well-supported parents are key. Two aspects matter here. First, clinicians should provide sufficient, personalized information and support to parents which do not only focus on autism’s downsides. This feeds in with recent calls to transform the diagnostic process and discourse in a neurodiversity-affirming way (32–34).

Second, adolescents hope their (non-autistic) parents are eager to learn more about autism, even though parents might start from a deficit-based, negatively connotated view of autism. Some evidence points out, for example, that when parents and clinicians serve as the main sources of knowledge about autism, as compared to online information, autistic adults are more likely to hold self-stigmatizing views (35). But according to Riccio and colleagues (36), parents can also play a large role in supporting a more accepting take on autism in their child. Sharing information about the autism diagnosis with one’s child in an open, voluntary and timely manner may foster more neurodiversity-affirmative views of autism. Delaying diagnostic disclosure until circumstances demand it^4^, or concealing the diagnosis for an extended period of time is associated with self-stigmatizing views of autism in adolescence (36). Interestingly, parents who are autistic themselves might light the way here as it seems that they are better positioned and more confident to inform their child in such affirmative ways, as they can build on their own experiences (37).

## Implications for the ethical debate on early autism diagnosis

In common descriptions of autism’s political history, autistic people and (non-autistic) caregivers of autistic children are often situated as opponents (38, 39). When introducing the ethical debate on early autism diagnosis, we also highlighted such potential opposition. We specifically touched upon the tension in applying the right to autonomy for caregivers and children, respectively. On the one hand, caregivers should be free to seek appropriate clinical care for their child as they see fit, including a diagnostic autism assessment. On the other hand, children should be protected against having important life choices determined by others before they can make those decisions themselves. This is sometimes referred to as children’s ‘right to an open future’ (8, 40). In certain cases, such important choices can and should be postponed in order to include the child in the discussion at a later age. Determining the risk of BRCA-related breast cancer is such an example. Since this disease only manifests in adulthood, the child in case has no benefit in early testing. Bioethicists and professional organizations therefore advise against childhood testing for this type of adult-onset conditions. However, other cases allow caregivers more liberty to exercise their right to make decisions, i.e. when information is clinically actionable and postponing action would harm the child. Newborn screening of metabolic disease such as phenylketonuria is such a textbook example.

More recently though, Garett et al. (41) have criticized the “right to an open future”-argument for its ambiguity and lack of nuance. How absolute should this “right” be interpreted and when are counterarguments sufficiently weighty to override it? What does “open” refer to? Is it about maximizing life options quantitatively? Or is it about keeping key qualitative opportunities open? Also, what if the case at hand is not about radically closing any options, but rather about altering the odds to take a future pathway?

Instead, Garett and colleagues (41) suggest to move from a right-not-to-be overridden toward an interest-based interpretation. They propose children have an interest in an open future (i.e. an interest to have their developing autonomy respected), but this is an interest to be weighed against many others including access to health and healthcare, protection from neglect and abuse, and developing one’s identity. “In other words, an open future is best understood not as a separate principle of pediatric bioethics, but instead as one component of its traditional focus on interests and balancing benefits and harms to children and family” (p. 2193, ibid).

If we were to apply this interest-based approach to our case of early autism diagnosis, then this interview study clearly contributes to the empirical dimension of balancing the benefits and harms from the perspective of autistic youth. We would certainly welcome ethical analyses using our study results in this way. However, our findings maybe even go one step further as they do not simply stress autistic youth’s variety of interests on top of merely respecting (developing) autonomy. Our interviewees showed that they went beyond the binary of parents’ versus children’s individual interests, as if these were two entirely separate issues that can be balanced against one another. Rather, we observed that adolescents occupied fairly generous positions toward their parents and stressed the relevance of a timely diagnosis for the *relationship* between parents and children. This clearly contrasts the political history of autism, it contrasts previous theoretical outlines of the debate on early autism diagnosis, but also differs from an interest-based interpretation of the “open future”-argument (7, 8, 41). Throughout the interviews, we did not come across such opposition between adolescents and their caregivers. Many acknowledged the difficulties of raising a child developing atypically and showed an understanding of parents’ choices to initiate a diagnostic assessment to get more grip on the situation and to learn how to improve their interaction with their child.

Moreover, adolescents’ remarks on how their diagnostic label functions as a service entry ticket, as a broker of understanding, and as justification for autism-adapted parenting styles converge with established findings from the qualitative literature on parenting autistic children (42).

In a previous interview study on the same topic with parents of young (potentially) autistic children, we described the complexity caregivers experience in ‘navigating the ab/normal binary’ (6). Similar to the adolescents in this current study, parents weighed potential benefits against risks, for example, in choosing when and to whom to disclose their child’s diagnosis with the risk of invoking stereotypical, negative or incorrect views on the part of their listeners. In that parent interview study, we discussed the context-dependency of valuing a timely autism diagnosis. For example, some parents indicated that an autism diagnosis for a second or third autistic child in their family was less urgent than for the first one, as they already had relevant knowledge and accommodations put in place to serve certain autistic needs. This finding echoes the lesser added value some of the interviewed adolescents attributed to their diagnosis when they felt already well-accommodated.

Of course, even when stressing the entanglement of interests of autistic youth and their (non-autistic) caregivers, are not entirely identical. And we certainly want to avoid overshadowing the experiences of autistic youth by those of their parents (18). Yet, we find it noteworthy that “experiences of neurodivergency” seem to expand beyond the bodies and minds of neurodivergent people themselves, as close relatives such as parents also live through (part of) the social and societal pressures of neuronormativity. This way, our findings empirically illustrate theoretical descriptions of disability -and by extension, neurodivergency- as fundamentally *relational* phenomena. Disability theorist Alison Kafer for example has put this as follows:

Friends and family members of disabled people are often affected by ableist attitudes and barriers, even if they are not themselves disabled. (…) not only does disability exist in relation to able-bodiedness/abled-mindedness, such that disabled and abled form a constitutive binary, but also, to move to a different register of analysis, disability is experienced in and through relationships; it does not occur in isolation. My choice of a relational model of disability is intended to speak to this reality (43, p. 8).

### Relations, rather than rights

Taking this altogether, it seems less interesting to take a purely rights-based ethical approach aiming for answers applicable to all cases of early autism diagnosis. A more nuanced-interest based approach as suggested by Garett et al, would already capture more nuance. However, the importance of fostering *relations* beyond individual interests deserves adequate attention, in our view. Therefore an explicitly relational and contextualized ethical approach, such as a (disability-informed) care ethics seems fruitful here (44, 45).

As our findings show, obtaining and mobilizing an autism diagnosis is not considered either *good* or *bad* in and of itself. The value of a timely diagnosis depends on whether it is being put to good use to meet needs in a given context. As argued by some of our interviewees, sometimes their context is already fairly accommodating, which makes the need for a formal diagnosis less pressing. But in the cases when a relatively early diagnosis was considered favorable, the crux lies in how the label is mobilized afterward. Here, autistic adolescents necessarily depend on their caregivers and clinicians. Such dependency relationships are well theorized by care ethicists. Specifically, the care ethics notion of “nested dependencies” might be helpful here. Nested dependencies refer to the reality that those who provide care are also dependent on others, in turn, to sustain their care work, creating webs of interdependence (46, 47). Adolescents pointed this out too: caregivers need to be adequately supported to make early diagnostics valuable.

Right here, in the support of parents, we believe the ethical tension described above can be overcome: young, autistic children can indeed not voice their opinion on early diagnosis and on appropriate post-diagnostic care. But in contrast to infants and toddlers, autistic adolescents and adults surely *can* join the conversation in name of the autistic community in general. Of course, such community consultation is no full replacement for involving individuals in personal healthcare decisions, but it does ease the ethical tension by adding autistic voices to the debate which have been largely missing so far.

One such valuable contribution by autistic adults are the lessons to be learnt from autistic parents disclosing an autism diagnosis to their child in a spontaneous and joyous way, setting the scene for a more accepting take on autism (36, 37). In the same vein, recent work has begun documenting the views of autistic adults regarding priorities in early autism care. Reducing autism characteristics systematically scores low, for example, but supports that increase communication skills -in whatever shape or form- are rated favorably, as this is considered an autonomy-enhancing early intervention (48–50). Such autistics-informed guidance might help caregivers and clinicians to make the best out of an early diagnosis even though the autistic young child in case cannot voice their opinion yet.

## Strengths and limitations

For this interview study, we relied on recruitment via an established database of a diagnostic center. Compared to convenience sampling methods via social media or advocacy groups, we believe to have included a sufficiently diverse sample. We excluded participants with a moderate and severe intellectual disability, but it was possible to include interviewees with a borderline IQ, a group which is often excluded too in similar research (17). Adolescents who participated via videocall reported that it felt less socially intrusive than a home visit and using a headset while video calling avoided distraction from other stimuli in the room. Providing the topic list beforehand and visualizing this during the interview were also appreciated by participants.

Despite our efforts at increasing accessibility, we have also noticed the limitations of the classical in-depth interview methodology in capturing the experiences and opinions of those adolescents in the lower range of borderline IQ scores. This also has to do with the nature of our research and interview questions requiring a certain metacognitive reflection on something abstract as an (early) autism diagnosis. Future research will need to continue engaging with questions of inclusivity and pursue ongoing innovations to make qualitative methodologies more accessible for participants across the entire autism spectrum, including those with below-average intellectual and verbal communication abilities (51–53).

## Data Availability

The raw data supporting the conclusions of this article will be made available by the authors, without undue reservation.

## Footnotes

1 We will use identity-first language in this manuscript (e.g. ‘autistic adolescents’). The rationale for this choice is twofold. First, it is the option preferred by a majority of English-speaking autistic people in Western countries queried on the topic (Keating, 2022); and second, with this choice we aim to contribute to the shift towards understanding autism as a social identity, rather than merely as a developmental disorder. We have retained person-first language in quotes as they occurred in the interviews. This way we want to do justice to our participants’ own words in Dutch and to illustrate diverging opinions in Dutch-speaking regions, where identity-first language is a minority preference. Where possible, we opted to use “autism” instead of “autism spectrum disorder” or “ASD” since the latter two options imply an inherent coupling of autistic traits to distress or pathology. To the contrary, the term “autism” provides more space to capture the wide set of autistic lived experiences that participants shared with us, whereas the clinical term “ASD” is more strictly delineated by its diagnostic criteria.

2 In the academic literature, ‘early’ autism detection and diagnosis often refer to identifying children under the age of three years (4). When we discuss ‘the ethics of early autism diagnosis’ in this paper, it is also this age group of infants and toddlers up to 36 months of age we have in mind. Clinically and colloquially, ‘early’ diagnosis is, however, used more loosely. Therefore, during recruitment and in our interviews, we sometimes used different cut-off points to demarcate ‘early’. When this occurs, we will clarify in the text as we go.

3 Also, ‘explaining’ should not be interpreted here necessarily as causally explaining something, in its scientific sense. Such causal claims would obviously run into the conceptual fallacy of ‘reification’, mixing up the construct of the diagnostic label with the ‘actual’ and ‘underlying’ pathways that shape autistic behaviour (as far as these exist in any knowable way). It is a circular argument when we first consider autistic behaviors as criteria to assign the autism diagnostic label, to then use this label to explain these very same behaviors.

4 In our study, we came across at least one clear example of such forced disclosure. At the age of 12, one boy learned he was autistic as he saw his mother handing over a document mentioning the diagnosis while registering for high school. The adolescent described this as an unsettling experience.
