# Supplementary material for: Getting the timing right. Autistic adolescents reflect on the value of an early diagnosis
Source: Front Psychiatry. 2026 May 14;17:1735842. doi: 10.3389/fpsyt.2026.1735842 (PMC13217295; doi:10.3389/fpsyt.2026.1735842)
Supplement: Supplementary file 1 [file SupplementaryFile1.pdf]

Supplementary Material

Patient database

Participants were recruited via the database of the Expertise Centre for Autism embedded within the University Hospitals of Leuven, Belgium. This patient database comprises all persons that have obtained a diagnosis of an autism spectrum disorder (ASD) at the center, following the DSM-5 diagnostic criteria, or a DSM IV equivalent when diagnosed before 2013. The center is situated in an academic hospital but it has a wide appeal in the region. The center mainly focuses on diagnostic assessments for autism and low-frequency follow-up for care coordination. Listing in the database therefore does not imply that the person in case is currently in clinical follow-up.

Consent procedure

We sought adolescents' assent by first sending them the adolescent version of our study information brochure by either email or post. We invited them to read and discuss possible questions already with their parents. When meeting for the face-to-face interview or at the start of the video call, we took time to go over this information orally, step-by-step, and adolescents could ask questions at this point. Participating adolescents were offered two cinema ticket vouchers as compensation for their efforts.

IQ Range

For various reasons, we chose to present IQ ranges rather than IQ scores. First, these scores are several years old for most adolescents, as IQ testing sometimes dates back to the time of their autism diagnostic assessment. Also, total IQ is an imperfect summarizing outcome as it pulls together various, and potentially heterogeneous cognitive abilities. We did not have data on the age at which IQ was measured, the instruments used, or clinical information (e.g., cooperation during assessment) that might help interpret the results.

The main reason that we included IQ ranges, instead of leaving out IQ as a descriptor entirely, is to indicate the diversity of the interviewees in this respect. We find this important as below-average IQ scores are often an exclusion criterium in autism research. The downside of reporting IQ is that readers might consciously or unconsciously attribute different levels of credibility to the experiences and opinions of our interviewees. To buffer this potential effect in part, we only report the IQ ranges in Table 1, yet, we do not repeat those when citing individual adolescents, in contrast to choices made by other authors (Berkovits et al., 2019).

Educational context

For context, ‘special education’ comes in various shapes in Flanders. Relatively unique in our region is the ‘type 9’ special education which is autism-specific. Some of our adolescents also attended non-autism-specific special education designed for intellectually disabled students. Special education in Flanders exists both in a segregated and more integrated way. Integrated special education means that classes for autistic students, for example, are organized within a regular school. Yet, in our study, we did not ask adolescents to clarify their educational context beyond the regular/special education categories. In both the segregated and integrated types of special education, pupils explicitly discuss their autism in class. This group-based engagement with autism discourses in an autism-specific setting might have influenced interviewees responses to our questions. As such, this might be a regional factor impacting the findings.

Topic list

Before the first actual interview question, we asked which words the participant was used to and comfortable with to refer to autism. During the interview, we stuck then to the preferred terminology of the adolescent, instead of using ‘autism’ as indicated in the topic list below *(‘We know that you have been assigned a diagnosis of autism spectrum disorder. Some people rather speak about autism, ASD, Asperger’s or PDD-NOS. Which words do you prefer?’).* All adolescents indicated they used ‘autism’ or ‘ASD’, some added ‘Asperger’s’ as an additional option.

1. *If someone wants to learn more about autism via you, how would you explain autism?*
2. *Do you sometimes feel different compared to the people around you? How do you notice?*
3. *To what extent is autism part of who you are?*
4. *Do you think you were born with autism? What does that mean to you?*
5. *When did you learn you have an autism diagnosis?*
   *[*Follow-up questions: *Who told you? What was it like to hear this? If you were to tell your child they were autistic, what would be the right age to do so? What did you think about the age you were told you are autistic? Would you have preferred to have received your diagnosis earlier or later in time? Some favor very early diagnosis of autism, i.e. under the age of three year, what do you think about this?]*
6. *Do you experience autism differently in various contexts?*
7. *How do you see your future?*

When designing the topic list, we took into account potential pitfalls when engaging with autistic people in qualitative research. Fayette and Bond (2018) signaled that the power imbalance between researcher and participant might generate pressure to provide an exact, right answer to the questions asked. Moreover, abstract or future-oriented questions can be confusing. Therefore, we started the interview by explicitly stating that the questions are not like a test or exam, that there are no right or wrong answers and that it is no problem if they could not answer the question. For each question in the topic list, we prepared reformulations that were more concretely formulated in case the adolescent presented issues

understanding. Before starting the actual interview, we also questioned what the interviewee's preferred terminology was concerning the diagnosis, i.e. autism, ASD, autism spectrum condition, Asperger's etc. We did so to eliminate terminological confusion and to avoid offending the adolescent.

In the interviews with adolescents with a TIQ in the borderline range, we did not only put the topic list on the table, but also an additional sheet with visual representations of the main interview topics. With a separate paper arrow pointing to these visuals, we indicated the interview's progress. Some adolescents appreciated this, others only paid attention to the written topic list during the interview.
